# Supplementary material for: The hepatic transcriptome is differentially regulated by a standardized meal in healthy individuals compared to patients with fatty liver disease
Source: PLoS One. 2025 Jun 9;20(6):e0307345. doi: 10.1371/journal.pone.0307345 (PMC12148195; doi:10.1371/journal.pone.0307345)
Supplement: S1 Table — (DOCX) [file pone.0307345.s001.docx]

S1 Table The 10 most up- and downregulated genes in NAFLD compared to healthy (fold change)

| **Gene symbol** | **fold change** | **adjusted p-value** | **GOBP** | **Gene name** |
| --- | --- | --- | --- | --- |
| *Upregulated in NAFLD* | | | | |
| PRKCE | 3.41 | 2.35791E-07 | apoptotic process | protein kinase C epsilon |
| ZBTB33 | 1.58 | 1.15955E-06 | regulation of transcription by RNA polymerase II | zinc finger and BTB domain containing 33 |
| COL5A3 | 7.06 | 1.0809E-05 | extracellular matrix organization | collagen type V alpha 3 Chain |
| PLIN1 | 2.91 | 1.0809E-05 | lipid metabolic process | perilipin 1 |
| TBX15 | 2.79 | 1.0809E-05 | regulation of transcription by RNA polymerase II | T-box transcription factor 15 |
| TP53INP1 | 1.94 | 1.0809E-05 | apoptotic process | tumor protein p53 inducible nuclear protein 1 |
| TSPAN3 | 2.29 | 1.28328E-05 | biological process | tetraspanin 3 |
| SERPINE1 | 6.72 | 1.5757E-05 | negative regulation of plasminogen activation | serpin family E member |
| LOC101927745 | 6.59 | 1.89337E-05 |  | uncharacterized LOC101927745 |
| ISM1 | 3.50 | 2.27326E-05 | negative regulation of angiogenesis | isthmin 1 |
| *Downregulated in NAFLD* | | | | |
| MET | 0.62 | 3.80107E-06 | signal transduction, cell surface receptor signaling pathway | MET proto-oncogene, receptor tyrosine kinase |
| FNDC3B | 0.55 | 4.05914E-06 | none | fibronectin type III domain containing 3B |
| RBMS1 | 0.58 | 5.65067E-06 | DNA replication, RNA processing | RNA binding motif single stranded interacting protein 1 |
| DIO3OS | 0.18 | 6.37569E-06 | none | DIO3 opposite strand upstream RNA |
| KRTCAP3 | 0.39 | 8.9979E-06 | none | keratinocyte associated protein 3 |
| IGSF8 | 0.74 | 1.0809E-05 | cell motility | immunoglobulin superfamily member 8 |
| RPL5 | 0.72 | 1.0809E-05 | translation | ribosomal protein L5 |
| GAS5 | 0.66 | 1.0809E-05 | cellular response to oxidative stress | growth arrest specific 5 |
| HSD17B2 | 0.57 | 1.0809E-05 | lipid metabolic process | hydroxysteroid 17-beta dehydrogenase 2 |
| PACSIN3 | 0.49 | 1.46347E-05 | cytoskeleton organization | protein kinase C and casein kinase substrate in neurons 3 |
