# Supplementary material for: The hepatic transcriptome is differentially regulated by a standardized meal in healthy individuals compared to patients with fatty liver disease
Source: PLoS One. 2025 Jun 9;20(6):e0307345. doi: 10.1371/journal.pone.0307345 (PMC12148195; doi:10.1371/journal.pone.0307345)
Supplement: S2 Table — (DOCX) [file pone.0307345.s002.docx]

**S2 Table** Gene list of the most up-and downregulated genes in NAFLD vs cirrhosis and cirrhosis vs. NAFLD.

| **Gene symbol** | **fold change** | **adjusted p-value** | **Gene name** |
| --- | --- | --- | --- |
| **Cirrhosisvs. NAFLD** | | | |
| *Upregulated in cirrhosis* | | | |
| GSN | 3.78 | 1.85468E-19 | gelsolin |
| CHI3L1 | 39.74 | 2.51258E-14 | chitinase 3 like 1 |
| EFEMP1 | 10.32 | 2.63548E-14 | EGF containing fibulin extracellular matrix protein 1 |
| ITGBL1 | 7.73 | 9.40364E-14 | integrin subunit beta like 1 |
| JAG1 | 3.27 | 3.18541E-13 | jagged canonical Notch ligand 1 |
| ITGA2 | 7.89 | 8.51962E-13 | integrin subunit alpha 2 |
| CCL19 | 12.17 | 4.92699E-12 | C-C motif chemokine ligand 19 |
| GOLM1 | 4.73 | 5.20807E-12 | golgi membrane protein 1 |
| SOD3 | 5.29 | 9.72764E-12 | superoxide dismutase 3 |
| KRT19 | 5.62 | 1.95144E-11 | keratin 19 |
| *Downregulated in cirrhosis* | | | |
| POSTN | 0.11 | 4.11874E-23 | periostin |
| FAM151A | 0.05 | 7.08064E-17 | family with sequence similarity 151 member A |
| SMIM14 | 0.54 | 1.27505E-11 | small integral membrane protein 14 |
| NCAM2 | 0.06 | 4.95329E-11 | neural cell adhesion molecule 2 |
| HSD17B14 | 0.15 | 2.30936E-10 | hydroxysteroid 17-beta dehydrogenase 14 |
| FOLH1 | 0.43 | 4.39593E-10 | folate hydrolase 1 |
| RGS5 | 0.42 | 1.37362E-09 | regulator of G protein signaling 5 |
| LOC157273 | 0.36 | 1.7026E-09 | uncharacterized LOC157273 |
| CYP2C19 | 0.07 | 1.7026E-09 | cytochrome P450 family 2 subfamily C member 19 |
| NR1H3 | 0.61 | 2.8665E-09 | nuclear receptor subfamily 1 group H member 3 |
| **Cirrhosis vs healthy** | | | |
| *Upregulated in cirrhosis* | | | |
| GSN | 6.90 | 2.01926E-36 | gelsolin |
| COL4A4 | 4.89 | 5.72126E-24 | collagen type IV alpha 4 chain |
| ZBTB33 | 2.27 | 8.83997E-21 | zinc finger and BTB domain containing 33 |
| GOLM1 | 8.78 | 1.16953E-20 | golgi membrane protein 1 |
| ITGBL1 | 13.13 | 5.51609E-19 | integrin subunit beta like 1 |
| CHI3L1 | 70.65 | 6.19722E-17 | chitinase 3 like 1 |
| NFASC | 6.28 | 1.00431E-16 | neurofascin |
| GLIS2 | 6.78 | 1.26128E-16 | GLIS family zinc finger 2 |
| AEBP1 | 6.41 | 1.30256E-16 | AE binding protein 1 |
| LTBP2 | 7.20 | 4.98384E-16 | latent transforming growth factor beta binding protein 2 |
| *Downregulated in cirrhosis* | | | |
| POSTN | 0.12 | 7.49263E-20 | periostin |
| PACSIN3 | 0.22 | 9.04792E-19 | protein kinase C and casein kinase substrate in neurons 3 |
| HSD17B14 | 0.09 | 4.94316E-15 | hydroxysteroid 17-beta dehydrogenase 14 |
| RGS5 | 0.32 | 7.16337E-15 | regulator of G protein signaling 5 |
| CYP2C19 | 0.03 | 1.52553E-14 | cytochrome P450 family 2 subfamily C member 19 |
| DNASE1L3 | 0.49 | 6.84768E-14 | deoxyribonuclease 1 like 3 |
| FAM151A | 0.05 | 8.28694E-14 | family with sequence similarity 151 member A |
| ASXL3 | 0.12 | 9.85987E-14 | ASXL transcriptional regulator 3 |
| NR5A2 | 0.50 | 1.15035E-12 | nuclear receptor subfamily 5 group A member 2 |
| BLVRB | 0.51 | 3.469E-12 | biliverdin reductase B |
