# Supplementary material for: The hepatic transcriptome is differentially regulated by a standardized meal in healthy individuals compared to patients with fatty liver disease
Source: PLoS One. 2025 Jun 9;20(6):e0307345. doi: 10.1371/journal.pone.0307345 (PMC12148195; doi:10.1371/journal.pone.0307345)
Supplement: S1 Fig — (DOCX) [file pone.0307345.s003.docx]

**
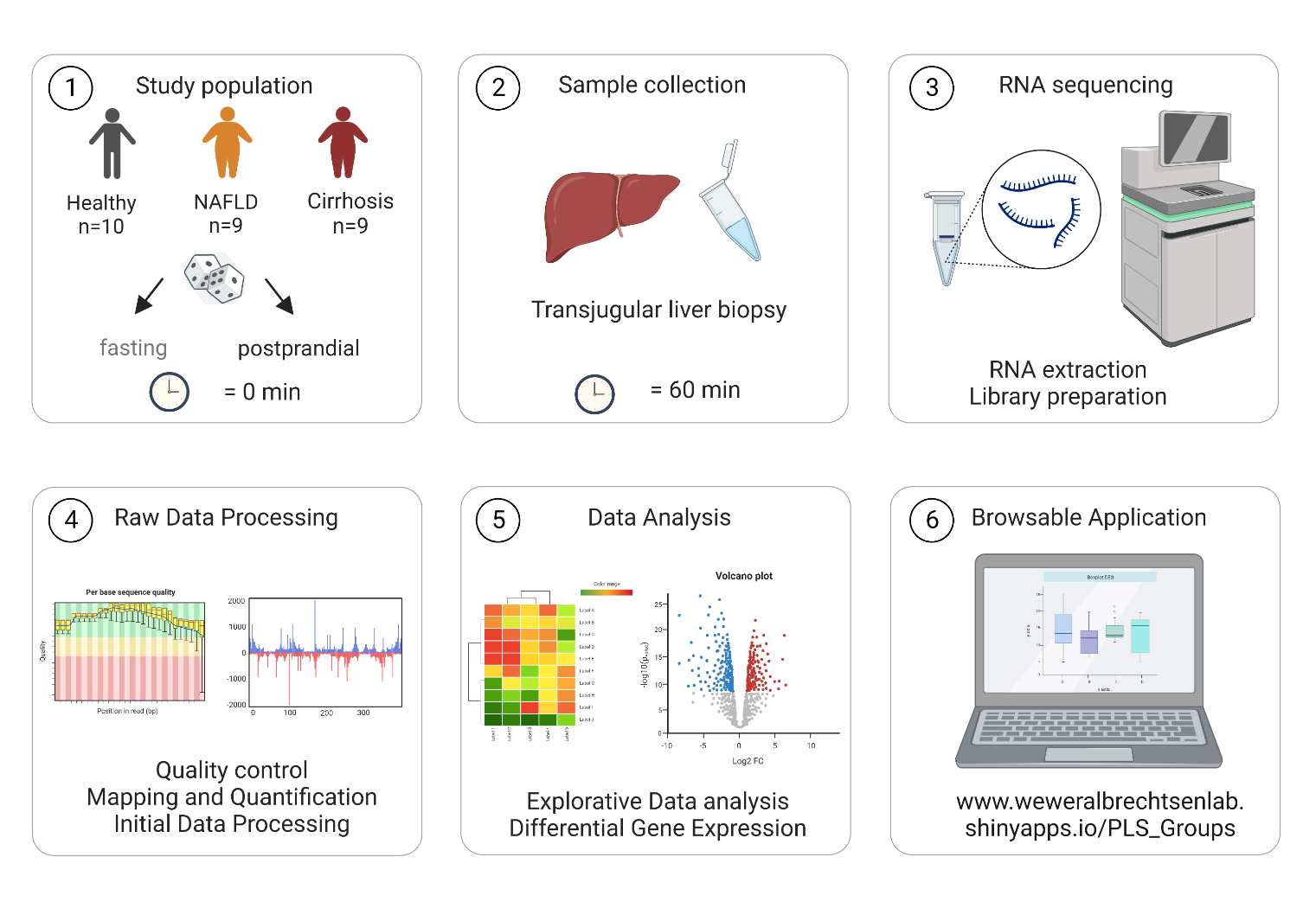
**

**S1 Figure.** 10 healthy controls, 9 patients with NAFLD and 9 patients with cirrhosis were randomized to fasting or postprandial at 0 minutes. 2) Liver biopsies were collected after 60 minutes. 3) We performed RNA sequencing with short-read Illumina sequencing technology. 4) Raw data processing was performed. 5) Explorative data analysis and differential gene expression analyses were performed. 6) We developed a browsable app for easy data access ([www.weweralbrechtsenlab.shinyapps.io/PLS_Groups](http://www.weweralbrechtsenlab.shinyapps.io/PLS_Groups)).
