# Supplementary material for: The hepatic transcriptome is differentially regulated by a standardized meal in healthy individuals compared to patients with fatty liver disease
Source: PLoS One. 2025 Jun 9;20(6):e0307345. doi: 10.1371/journal.pone.0307345 (PMC12148195; doi:10.1371/journal.pone.0307345)
Supplement: Attachment — Submitted filename: author response.docx [file pone.0307345.s005.docx]

Reviewer #1: The authors of a manuscript “PONE-D-24-26821: The hepatic transcriptome is differentially regulated by a standardized meal in healthy individuals compared to patients with fatty liver disease” present a study evaluating hepatic transcriptome at both fasting and postprandial states in patients with NAFLD, cirrhosis, and controls. They described huge number of differentially expressed genes (DEG) between healthy controls and patients with NAFLD and patients with cirrhosis. The authors also described that the hepatic transcriptome changed significantly during meal and the changes were blunted in patients with NAFLD and cirrhosis. They found an increase in pro-inflammatory and pro-fibrotic genes in NAFLD and cirrhosis. The main result is that the hepatic transcriptome is differentially regulated in healthy individuals compared to patients with NAFLD after meal.
Additionally, the authors developed a browsable resource enabling easy access to their data (two web-based applications).

**Author Response:** We appreciate the time and effort from the reviewer and also the constructive and positive comments. We have at our best revised the manuscript accordingly.

1. Healthy controls were markedly younger than patients (NAFLD, cirrhosis). Could the age influence the changes in transcriptome??

**Author Response:**  Yes. A recent study showed some genes are affected by age, see <https://pubmed.ncbi.nlm.nih.gov/34959291/>

The major changes between the groups are however not explained by age based on the genes affected (comparison of the current study with the literature mentioned).

2. What was the aetiology of cirrhosis – NAFLD??

**Author Response:**  There were no requirements regarding the etiology of cirrhosis, Child‐Pugh score, medication, or previous decompensation. Patients with ongoing alcohol abuse or malignant diseases were excluded. This has now been included to the revised manuscript.

3. Did the authors measure HVPG during liver biopsy? If yes, did they observe any differences in transcriptome in relation to severity of portal hypertension??

**Author Response:**  Yes, we did measure HVPG. We did not find any difference across in group. The HVPG were for healthy 2±0, for NAFLD 3±0 and for cirrhosis 11±2 mmHg.

4. The authors state that the presented study is one of the few studies including liver biopsies from healthy controls. Did the authors observe any complication during transjugular liver biopsy in healthy people?

**Author Response:**  No not a single one!

This is an interesting study describing the differences in transcriptome in healthy controls, patients with NAFLD and patients with cirrhosis both fasting and after meal.
The added value is the creation of functional web applications for data viewing and analysis.

**Author Response:** Thanks!

Reviewer #2: Summary
Grandt et al. investigated how food intake affects the hepatic transcriptome in patients with non-alcoholic fatty liver disease (NAFLD), cirrhosis, and healthy controls. Liver biopsies from these groups were analyzed to identify differentially expressed genes (DEGs) and gene ontology biological processes. Significant differences were found between the groups, with an increase in pro-inflammatory and pro-fibrotic genes in NAFLD and cirrhosis and a decrease in metabolic-related genes. In the presented experimental setting, food intake seemed to play a minor role in hepatic transcriptomes of healthy controls and diseased livers. The authors developed a web-based resources to make the data easily accessible.

General comment
Although the manuscript is structured and written comprehensibly, it lacks a clear presentation of scientific hypotheses. Clarifying the purpose of the study would strengthen the use of the online resource, a strong point of the study. Another positive aspect is the sampling via biopsies rather than post-mortem tissue collection, crucial for capturing rapid changes in liver metabolism and RNA expression. However, a biopsy only represents a small part of the whole organ, which could potentially lead to misleading results by missing disease-relevant regions and cell types. To mitigate this limitation, the authors could utilize publicly available (single-cell) datasets to put their findings in a broader context. Additionally, it would help to add histological assessments of the biopsies and validate some of the genes in situ or by PCR. If food intake does not drive significant transcriptional changes in the present experimental setting, this should also be reported and discussed.

**Author Response:** Thank you for your time and effort and constructive inputs.

We have done our best to revise the manuscript in line with your comments. We have not included publicly available dataset. All our data have been made publicly available and browsable for readers.

Specific points

1. Please state clear hypothesis and idea: Clearly articulate the hypotheses and underlying ideas behind the study to strengthen the concept and manuscript. The phrases "Our aim was to investigate the hepatic transcriptome at both fasting and postprandial states in patients with..." or "Next-generation sequencing (NGS) provides a hypothesis-free approach with high specificity and sensitivity allowing insights into molecular mechanisms by identifying crucial genes..." are insufficient. Although the approach is conceptually "hypothesis-free," it is crucial to convey what insights or applications are anticipated from the data.

**Author Response:** Revised accordingly. We hope this is sufficient and aligns with the comment by the reviewer.

2. Clarify transcriptional changes: The statement "The hepatic transcriptome changed significantly during a standardized meal and these changes were blunted in patients with NAFLD and cirrhosis" needs clarification. Transcriptional changes between fasting and postprandial states appear minimal (PCA, number of DE genes). Please clarify this or present new data/analyses to substantiate the claim.

**Author Response:** In table 3 and in the results section, we have provided details on which genes are affected differently between disease state.

3. The number of DE genes appears high for some comparisons (e.g., cirrhosis samples). Ensure that potential batch effects were analyzed and corrected; if so, provide insights. Additionally, validating detected genes using orthogonal methods (in situ hybridization, immunohistochemistry, RT-PCR) would strengthen the biological message.

**Author Response:** Thank you for this point. The changes align with literature. We have carefully performed the analysis in batches to circumvent such effects.

We have not performed additional verification as this does not align with any literature on proteome analysis – or genomic for that sake. Sorry!

https://www.nature.com/articles/s41591-022-01850-y

4. Provide histology of biopsies: Include histological assessments of the biopsies (“Part of the liver tissue was used for histological assessment”). This will help correlate histological findings with transcriptional changes.

**Author Response:** The NAS scores are shown in table 2.

5. Broader context: Integrating the present data into publicly available single-cell datasets would improve the study by providing context for transcriptional and potentially cellular changes in liver diseases. For example, see publications: DOI: 10.1016/j.xgen.2023.100301, DOI: 10.1016/j.isci.2021.103233)

**Author Response:**  We have included the two references in the revised manuscript.

6. Table 1: Please provide critical values/diagnostic thresholds, e.g. by using color green=normal vs red=above/lower than threshold. There might be regional differences in clinical standards/critical values.

**Author Response:**  Good point. However, the references are internationally used. If we are allowed by the editor, we can provide color coding.

7. A visual comparison of overlapping and non-overlapping DEGs would aid data presentation and evaluation. Consider using a Venn diagram.

**Author Response:**  The DEG are shown in table 3.

8. PCA Loadings: Including PCA loadings could help describe differences between conditions. Consider adding the most informative loadings to the manuscript.

**Author Response:**  Thank you. We have two panels one showing differences between conditions and one between fasting an postprandial. We hope this is in line with your suggestions.
